# Supplementary material for: Low Expression of UBE2Z, a Target Protein of miR-500a, Is Associated with Poor Prognosis in Triple-Negative Breast Cancer
Source: Int J Mol Sci. 2025 Dec 29;27(1):361. doi: 10.3390/ijms27010361 (PMC12785900; doi:10.3390/ijms27010361)
Supplement: Supplementary file 1 [file ijms-27-00361-s001.zip › ijms-4023546-supplementary.pdf]

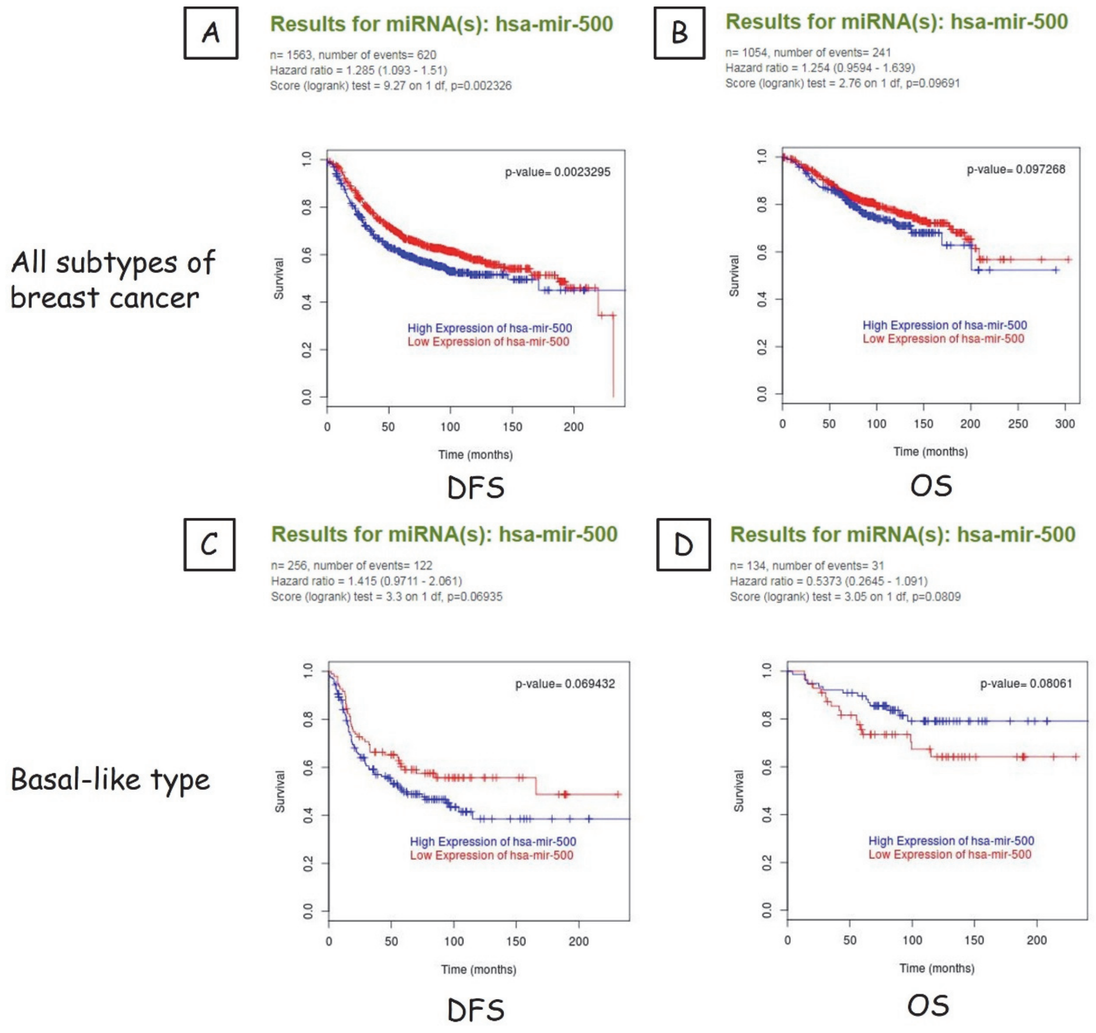

Supplementary Figure S1. Survival analysis of breast cancer patients using the BreastMark database. Association between miR-500 and disease-free survival (A) and overall survival (B) in all breast cancer patients. Association between miR-500 and disease-free survival (C) and overall survival (D) in triple-negative breast cancer patients.

Supplementary Table S1. Predicted target genes of over-expression miRNAs

MicroRNA ID	total	Predicted target genes
miR-500a-3p miR-501-3p miR-502-3p	13	UGT2B10, IBA57, PSMG1, MTHFD2, ZNF460, SOD2, ELAVL2, NCAPG2, C8orf33, EFCAB11, NAA30, ZBTB43, AGBL5
miR-500a-3p miR-501-3p	2	DENND4B, FDXR
miR-500a-3p miR-502-3p	2	COMMD4, RTFDC1
miR-501-3p miR-502-3p	42	TNIP3 KCNJ6 RBM3 LRRC58 TRIM72 CDC27 CDK6 PEG10 PLEKHB2 CSRP1 WSB1 MRRF TBL1XR1 NLGN4X SIGLEC15 COL23A1 SOCS7 CYP4F11 TLCD2 OIP5 CRK ENTHD1 MANBAL PLCB1 SLC2A12 SNRPD3 CA12 TRDN NPTXR STARD7 SMIM13 KMO GTPBP2 LYSMD3 IL21R TMEM135 RPL4 MSI1 SSTR1 FRS2 FCN2 EMP2
miR-500a-3p	85	C11orf31C11orf31 TMEM239 OTUD7B VIPR1 STRN3 ZNF695 UBE2Z FKBP9 BAZ2B C18orf25 C11orf24 PRR14L TAF8 CCDC113 ZFP62 MYLIP MRPS10 RPH3AL ORAI2 MCF2L2 SLC33A1 SLC7A11 DERL2 EMC8 NWD1 BMP8A MIXL1 SMARCA5 PAQR3 ALG14 TNFAIP2 FYN TRIM49C CERK IER3IP1 MYO1H KIAA1586 CCNT2 PRRC2B ANKEF1 OCIAD2 SNRPD1 CHMP2A PGM3 LRRC55 TNFRSF13C POLQ SOAT1 ZBTB46 PRRG4 SIGLEC8 GM2A CCDC171 VAV3 LAMB1 SH2B3 ZNF277 MRPS16 MUC21 PARD6B FADS2 PLA2G16 SPRY1 YIPF4 ANKRD27 MDM2 PAK2 FKBP15 RAN KIAA1958 KLHL15 DST PNO1 TOR1B HIF1AN FFAR4 MTPN F2RL3 CCDC47 PPP1R37 ZADH2 ZBTB8B CTDSPL RAB3D OPA3
miR-501-3p	4	UBE2H, FAM219B, MCM2, MCM4
miR-502-3p	8	ZSWIM6 ASXL2 MGAT5 DCTN2 AIM1 B4GALT5 PITRM1 RPS27

Supplementary Table S2. Predicted target genes of under-expressed miRNAs

MicroRNA ID	total	Predicted target gene
miR-6798-5p miR-7150	6	BTG2 DPH2 TMEM109 ATG2A KIAA1715 CC2D1B
miR-6798-5p	79	ITM2C QSOX2 SYT13 OLFML2A GFER SAP18 CALML3 FAM117B HYOU1 RNF157 SSTR3 BAZ2A PHF21B DTX2 OTUB1 ZFP36 CDCA4 POC1A FGFR4 ATXN7L3 MICB SETD1A NPBWR1 SESN3 DNLZ SUPT16H TNPO2 E2F1 SUSD2 LY6K ELOF1 NFASC LY6E THBS2 CCL22 LMBR1L TIGD5 MAPK13 ZFP91 WAC ADCY9 PARS2 FBXO2 PNMAL2 VAV3 GIGYF1 RAB15 SMC1A TMPRSS6 BCL2L2-PABPN1 KIF6 LGSN ZBTB8A KDM5C TACC3 NDRG3 PTBP1 ZBTB40 TAGLN2 ADO MAP3K9 VWA1 SPRED3 WEE1 KLF16 NLGN2 MIB2 NCR3LG1 SALL2 STK35 FN3K PSMB9 HMGB1 PABPN1 TOR2A NRF1 CRTC2 GABARAPL1 DDX39B
miR-7150	96	ACER2 DPM2 TAF1D APCDD1 AMER2 NKPD1 TUBD1 PPM1H ZNF185 SYNGR2 ZNF551 DYNLL2 ESYT1 RAC1 TBC1D4 SUMO1 IFNAR2 TP53 TOMM20 ARSK SATB2 PDE3A MALT1 GABRB1 CREBRF WDR13 ARL6IP4 ALKBH5 ZXDB TBL1XR1 GNL1 CREBZF ASB16 EDAR STOML3 SERPINH1 UNC13D NAV1 TEAD1 SYNM CD84 EPHB2 ETV5 KDM5A LBX1 WNT7B CHSY1 MAP1LC3B PITPNM3 RPL37A PPP1R11 TGOLN2 DDX19B KRT8 BMP3 S100A2 PHF19 KIAA0513 SIRPG RAI1 DCUN1D3 PPP1R15B CDNF ZNF710 SLC18B1 TNRC6A DPYSL5 ASXL1 C9orf62 ELP6 NFIX FAM73A ERCC6L APOPT1 SAR1B ATXN1L CRTAP EIF3L LONRF2 PFN1 EEF1A1 GPR156 MIDN EXOSC2 CPE PPP1R12A DHTKD1 HDLBP NSUN4 PHF15 PIM1 TPI1 NECAP1 TAB1 RBM23 HAND1

Supplementary Table S3. Relapse-related hub proteins predicted using the STRING algorithm

Gene ID	Function
RAC1	Plasma membrane-associated small GTPase
PAK2	Serine/threonine protein kinase
FYN	Non-receptor tyrosine-protein kinase
VAV3	Exchange factor for GTP-binding proteins RhoA, RhoG
EPHB2	Receptor tyrosine kinase
MIB2	E3 ubiquitin-protein ligase
UBE2Z	Ubiquitin-conjugating enzyme E2 Z
PSMB9	multicatalytic proteinase complex
EEF1A1	GTP-dependent binding of aminoacyl-tRNA to the A-site of ribosomes
RPL4	L ribosomal proteins
RPS27	Component of the small ribosomal subunit
SMARCA5	SWI/SNF-related matrix-associated actin-dependent regulator of chromatin subfamily A member 5
BTG2	Anti-proliferative protein
TP53	tumor suppressor
SUMO1	Ubiquitin-like protein
MDM2	E3 ubiquitin-protein ligase
SNRPD1	Small nuclear ribonucleoprotein Sm D1
PABPN1	3′-end formation of mRNA precursors
